# The Use of Iron-Doped Anatase TiO_2_ Nanofibers for Enhanced Photocatalytic Fenton-like Reaction to Degrade Tylosin

**DOI:** 10.3390/molecules28196977

**Published:** 2023-10-08

**Authors:** Xiao Wang, Wei Lu, Shangui Zhang, Changqing Guo, Kai Yang, Yan Sun, Yashi Shao, Qiyuan Li, Mingsheng Bu, Lianfeng Wu, Bo Wang, Dongjiang Yang

**Affiliations:** 1 State Key Laboratory of Marine Coatings, Qingdao 266071, China; zhangshangui@sinochem.com (S.Z.); wulianfeng@sinochem.com (L.W.);; 2 Marine Chemical Research Institute Co., Ltd., Qingdao 266071, China; 3 School of Environmental Science and Engineering, Qingdao University, Qingdao 266071, China; d.yang@qdu.edu.cn

**Keywords:** TiO_2_, Fe-doped, Fenton-like, photocatalyst, tylosin

## Abstract

The removal of antibiotics from wastewater to prevent their environmental accumulation is significant for human health and ecosystems. Herein, iron (Fe)-atom-doped anatase TiO_2_ nanofibers (Fe-TNs) were manufactured for the photocatalytic Fenton-like decomposition of tylosin (TYL) under LED illumination. Compared with the pristine TiO_2_ nanofibers (TNs), the optimized Fe-TNs exhibited improved visible-light-driven photocatalytic Fenton-like activity with a TYL degradation efficiency of 98.5% within 4 h. The effective TYL degradation could be attributed to the expanded optical light absorption and accelerated separation and migration of photogenerated electrons and holes after the introduction of Fe. The photogenerated electrons were highly conducive to the generation of active SO_4_^•−^ radicals as they facilitated Fe(III)/Fe(II) cycles, and to oxidizing TYL. Moreover, the holes could be involved in TYL degradation. Thus, a significant enhancement in TYL degradation could be achieved. This research verifies the use of iron-doped anatase nanofibers as an effective method to synthesize novel photocatalytic Fenton-like catalysts through surface engineering for wastewater remediation.

## 1. Introduction

Antibiotic accumulation in water environments could produce drug-resistant bacteria and resistance genes, posing great threats to ecosystems and human health [1,2]. Therefore, efficient and environmentally friendly measures are urgently needed to remove antibiotics before they are discharged into natural water environments. Traditional biochemical treatment has limitations in treating antibiotics in wastewater due to its inhibitory effect on microbial growth [3]. Through physical treatment methods such as adsorption, the membrane could concentrate and transfer pollutants, but they face the problem of further processing to completely eliminate contamination [4,5]. Fenton-like technology based on advanced oxidation processes (AOPs) has been demonstrated to be an efficient strategy to mineralize antibiotics by reacting with the produced reactive oxygen species [6,7,8,9,10]. Furthermore, the light-assisted photocatalytic Fenton-like reaction approach, combining the advantages of photocatalysis and Fenton-like reactions, shows great promise in the elimination of antibiotic pollutants in wastewater [11,12].

Heterogeneous Fenton-like photocatalysts play a decisive role in this oxidation process [13,14,15,16]. TiO_2_, possessing a band gap of about 3.0–3.2 eV, has been extensively investigated as a photocatalyst to degrade pollutants because of its bio-safety, low price, and chemical stability [17,18]. In particular, one-dimensional TiO_2_ nanofibers (TNs) have better photogenerated charge separation and transport performance than nanoparticles, leading to higher photocatalytic activity [19]. However, the critical problems, such as a limited light absorption edge, and massive charge carrier recombination and deactivation, need to be resolved to achieve high activity [20]. Thus, researchers have attempted to modify TiO_2_ with the purpose of inhibiting the recombination and deactivation of charge carriers or enhancing the response to visible light of TiO_2_ [21,22]. Among various types of modification methods, doping a transition metal into TiO_2_ is a good strategy. The stabilized M-O(OH) structure and the interaction between transition metal atoms and TiO_2_ could promote catalytic activity [23]. In addition, Fe is considered an environmentally friendly element in wastewater purification treatment. Fe atom-doped substrates could catalyze oxidizing agents such as H_2_O_2_ and peroxymonosulfate (PMS) to produce reactive oxygen species, which possess strong oxidation capabilities to mineralize contaminants [24,25,26,27,28,29]. Yin et al. [30] developed Fe-doped carbon-based catalysts for boosting Fenton-like reactions. Guo et al. [31] doped Fe atom-modified g-C_3_N_4_ and discovered that different kinds of pollutants could be degraded by photocatalytic Fenton-like reactions driven by visible light. Thus, the coupling of Fe atoms and TiO_2_ is assumed to be an appropriate approach to obtain photocatalytic Fenton-like catalysts with high efficiency to degrade antibiotic pollutants by adjusting the visible-light absorption range and photoinduced charge transfer.

Herein, Fe atom-anchored anatase TiO_2_ nanofibers (Fe-TNs) were prepared for photocatalytic Fenton-like antibiotic tylosin (TYL) degradation driven by white LED illumination. Compared with the pristine TiO_2_, Fe-TNs obviously displayed enhanced TYL degradation performance. TYL could be degraded by 98.5% within 4 h for optimal Fe-TN catalysis. The confined Fe atom on TiO_2_ could extend the visible-light absorption region and increase the charge transport efficiency, guaranteeing that more photoinduced electrons would be involved in the Fe(III)/Fe(II) cycle to facilitate PMS activation. And the formed active species could accelerate TYL degradation. Moreover, the photoinduced holes could participate in TYL degradation.

## 2. Results and Discussion

### 2.1. Structural and Morphological Characterization

**Figure 1:**
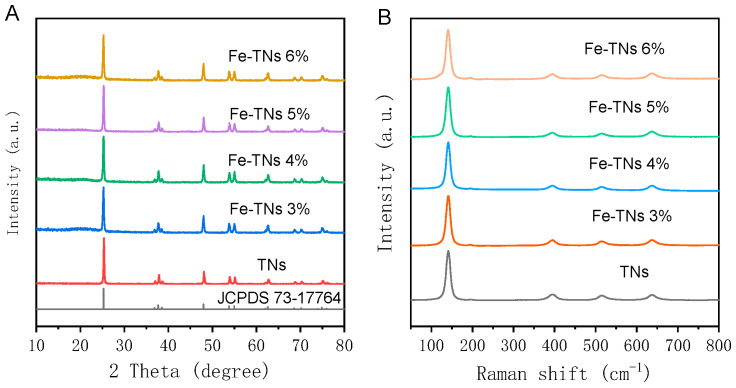
XRD patterns (**A**) and Raman spectra (**B**) of TNs and Fe-TNs with different iron amounts.

X-ray diffraction (XRD) patterns and Raman spectra were used to examine the crystalline forms of TNs and Fe-TN catalysts prepared with varying iron loading amounts. According to the XRD results shown in Figure 1A, the TNs and Fe-TN catalysts all displayed typical diffraction peaks at 25.28°, 37.80°, 48.05°, 53.89°, 62.30°, and 75.03°, which are indexed to the (101), (004), (200), (105), (204), and (215) crystal facets of anatase TiO_2_ [32]. Diffraction peaks of Fe-related compounds were not found, suggesting that the crystalline form of TNs remains unchanged after Fe modification. Furthermore, the TNs and all Fe-TN samples revealed the same Raman spectra (Figure 1B). The Fe-TN samples had similar Raman spectra to the TNs, with bands centered at 141 cm^−1^, 394 cm^−1^, 514 cm^−1^, and 636 cm^−1^ corresponding to anatase TiO_2_ Raman vibration modes.

**Figure 2:**
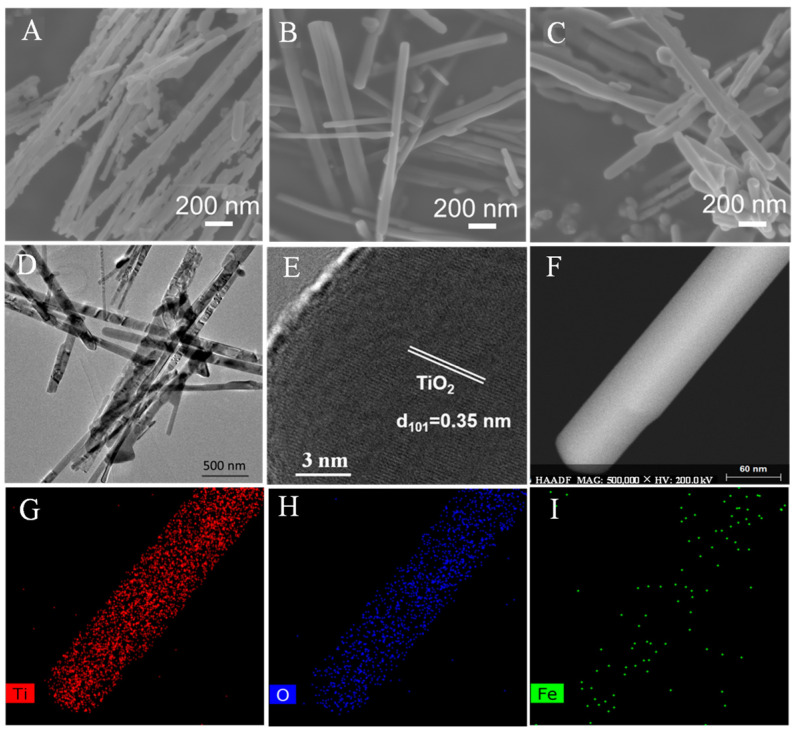
SEM images of (**A**) TNs and (**B**,**C**) Fe-TNs 5%. TEM (**D**–**F**) and EDX elemental mapping images (**G**–**I**) of Fe-TNs 5%.

The morphology and the elemental distribution of the produced TNs and Fe-TN catalysts were characterized via SEM and TEM coupled with energy-dispersive X-ray spectroscopy (EDX). Apparently, the TNs and all the Fe-TN samples appeared as a one-dimensional fibrous structures, as shown in the SEM (Figure 2A–C) and TEM (Figure 2D) images. The modification of TiO_2_ though Fe doping did not alter the one-dimensional fibrous structure. And the aggregation of crystalline-phase substances and Fe-related nanoparticles was not detected on the surface of the Fe-TNs, which was attributed to the dispersion of Fe at an atomic level. The lattice fringe with a spacing of 0.35 nm is due to the (101) plane of anatase TiO_2_ (Figure 2E). The N_2_ adsorption–desorption experiments suggested that the S_BET_ values of the TNs and Fe-TNs 5% were 26 and 18 m^2^/g. The introduction of Fe atoms slightly reduced the specific surface area (Figures S1 and S2). As shown in the HRTEM images (Figure 2F), the regular lattice fringe with a distance of 3.5 Å was attributed to the (101) crystal face of anatase TiO_2_. Moreover, Figure 2F and Figure S3 further confirmed that Fe-related nanoparticle aggregation was not detected on the surface of the Fe-TNs. The well-distributed elements of Ti, O, and Fe in the associated EDX elemental mapping images (Figure 2G–I) reveal the successful introduction of Fe on TNs. 

**Figure 3:**
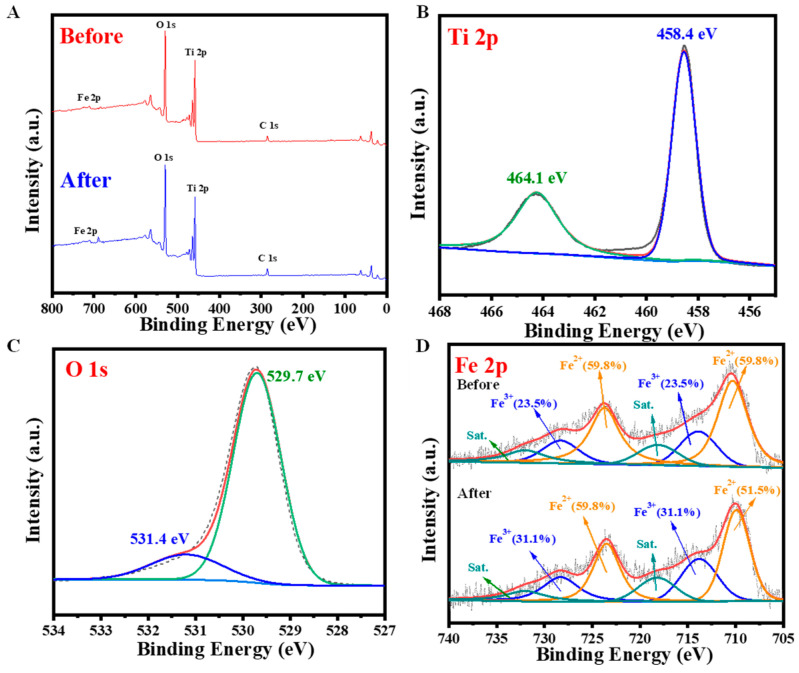
XPS spectra of Fe-TNs 5%. (**A**) Survey and high-resolution graphs of elements of (**B**) Ti 2p, (**C**) O 1s, and (**D**) Fe 2p before and after reactions.

The XPS full-scan spectrum (Figure 3A) of Fe-TNs 5% before and after the reaction shows the existence of Fe, Ti, O, and C elements in the Fe-TNs. The appearance of an Fe 2p signal in the Fe-TN sample proves the successful introduction of Fe into the TNs. The Ti 2p XPS spectrum (Figure 3B) consists of two major peaks at 458.4, 464.1 eV assigned to the binding energies of Ti 2p_5/2_ and Ti 2p_3/2_ of Ti^4+^, respectively. Both the surface adsorbed oxygen (O_ads_) and lattice oxygen (O_latt_) are clearly observed at binding energies of 531.4 eV and 529.7 eV in the Fe-TNs (Figure 3C). The Fe 2p spectra (Figure 3D) are deconvoluted into peaks centered at 713.6 eV and 728.3 eV for Fe 2p_3/2_ and Fe 2p_1/2_ of Fe(III), and the peaks at 710.3 eV and 724.1 eV for Fe 2p_3/2_ and Fe 2p_1/2_ of Fe(II). The fitted results suggest that the valence states of both Fe(III) and Fe(II) existed in the Fe-TNs. The proportions of Fe(III) to Fe(II) in Fe-TNs 5% after the reactions are higher than that before the reactions, indicating that Fe(II) was transformed into Fe(III) in the PMS activation process. The Fe(III)/Fe(II) cycle could realize PMS activation to generate radicals to degrade pollutants.

### 2.2. Tylosin Degradation Performance via Photocatalytic Fenton-like Reactions

**Figure 4:**
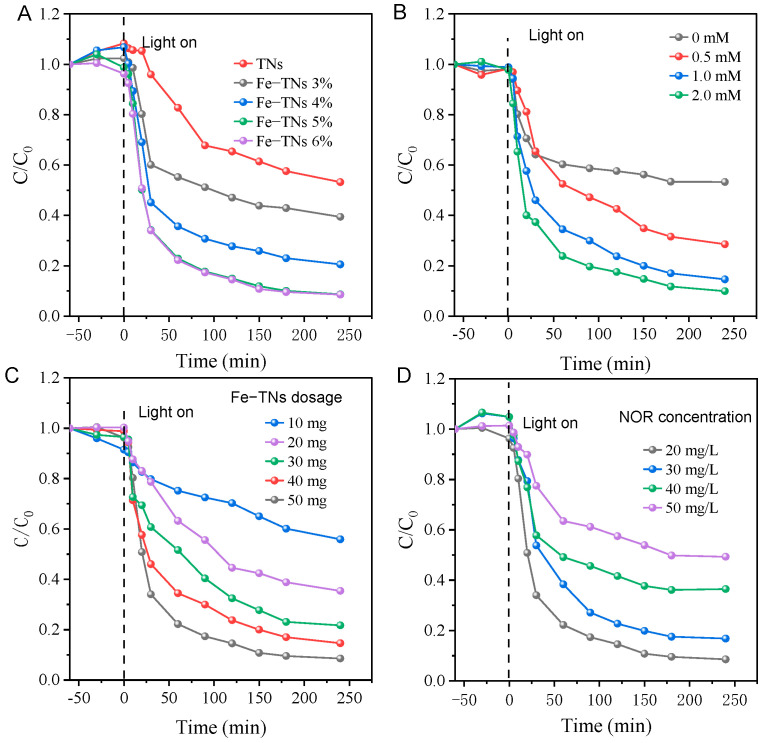
Photocatalytic Fenton-like degradation of TYL by TNs and Fe-TNs driven by visible light and the effect of the reaction parameter. (**A**) Fe loading amount, (**B**) PMS concentration, (**C**) catalyst dosage, (**D**) TYL concentration (conditions: 0.5 g/L catalyst, 20 mg/L TYL, 1 mM PMS unless otherwise stated).

The photocatalytic Fenton-like degradation of TYL by Fe-TNs in the presence of PMS was studied with TYL as the objective pollutant. The influence of Fe modification with different loadings on the degradation performance of TYL was investigated and the results are shown in Figure 4A. It shows that the Fe-TNs with 5% loading had the best performance in TYL degradation. And the degradation performance of Fe-TNs with 5% Fe loading was similar to those with 6% Fe loading, indicating that the further increase in Fe loading had no effect on TYL removal. The influences of PMS concentration, catalyst dosage, and TYL concentration on TYL degradation performance in this photocatalytic Fenton-like reaction catalyzed by TNs and Fe-TNs were separately investigated. As displayed in Figure 4B, as the PMS dosage increases, the TYL removal rate increased due to the generation of more SO_4_^•−^ species. Figure 4C shows that as the dosage of Fe-TNs increases, both the degradation efficiency and rate for TYL are significantly improved. As the TYL concentration increases, the degradation efficiency decreases (Figure 4D) because the photocatalyst’s limited active sites can be consumed by TYL by-products. Therefore, 0.5 g/L of Fe-TN catalysts and 1 mM PMS were chosen for the degradation of 20 mg/L TYL solution in the subsequent studies. Under these conditions, the optimized Fe-TNs 5% catalyst exhibits improved visible-light-driven photocatalytic Fenton-like activity with TYL degradation efficiency of 98.5% within 4 h, which is more advantageous than the other previous reported catalysts [13,14,20,33,34,35] (Table S1). Moreover, the TEM image (Figure S4) shows that there is no change in the morphology of Fe-TNs 5% after the reaction, indicating its structural stability.

**Figure 5:**
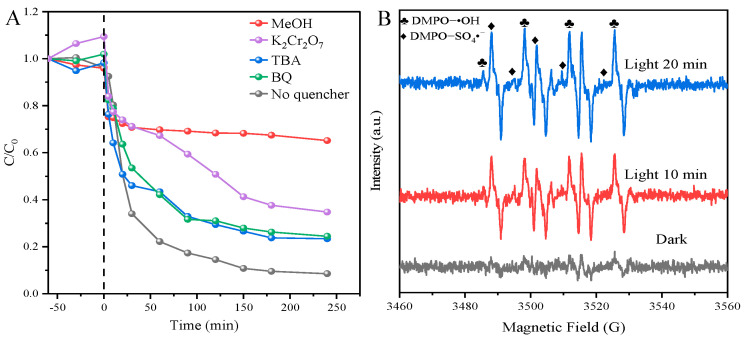
(**A**) Influence of different capture agents on TYL degradation (conditions: 0.5 g/L catalyst, 20 mg/L TYL, 1 mM PMS). (**B**) EPR spectra of DMPO adducts in this photocatalytic Fenton-like system catalyzed by Fe-TNs under the conditions of white LED light illumination and darkness.

The radical capture experiments were carried out to examine the primary reactive species involved in TYL degradation in this photocatalytic Fenton-like process catalyzed by Fe-TNs [36,37,38]. Methanol (MeOH) was utilized as an •OH and SO_4_^•−^ scavenger, and potassium dichromate (K_2_Cr_2_O_7_) as an electronic trapping agent. Tert-butanol (TBA) could quench •OH, and p-benzoquinone (BQ) could capture •O_2_^−^, •OH, and •O_2_^−^. As illustrated in Figure 5A, SO_4_^•−^, electrons, and •OH contributed to the degradation of TYL and SO_4_^•−^ and also play a significant role in this process. Furthermore, the electron paramagnetic resonance (EPR) spectra (Figure 5B) show that no radical signals appear under dark conditions. When PMS, catalysts, and LED light coexisted in the system, clearly, EPR signal peaks were presented, showing that radicals of SO_4_^•−^ and •OH could be produced under the condition of visible-light radiation.

**Figure 6:**
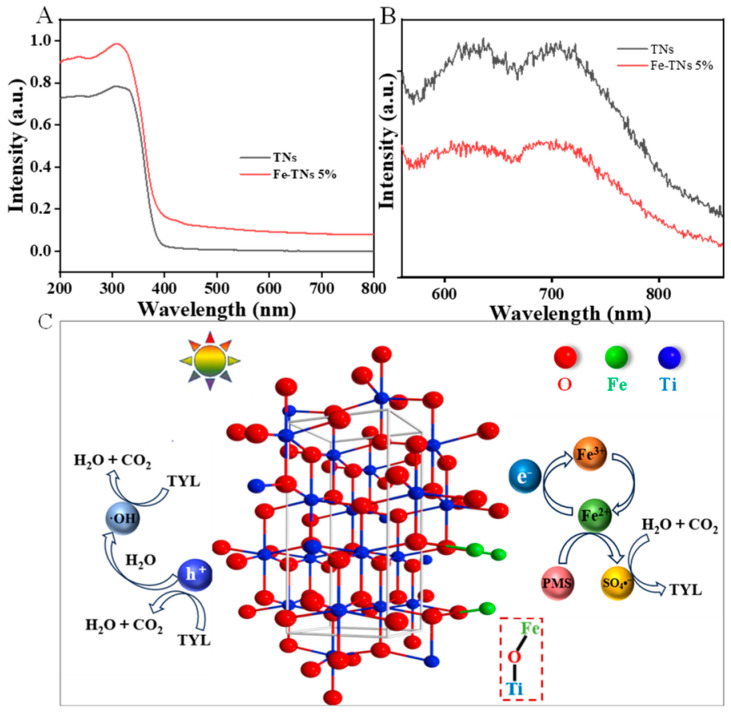
(**A**) UV–vis absorption, (**B**) PL spectra of TNs and Fe-TN catalysts, (**C**) proposed mechanism of TYL degradation over Fe-TNs.

The light absorption properties of TNs and Fe-TNs 5% were surveyed by assessing their UV-Vis absorption spectra (Figure 6A). Compared with TNs, the Fe-TNs revealed obvious extended and enhanced visible light absorption, proving that they promoted visible light absorption capacity after the introduction of Fe atoms. Based on the Tauc plots shown in Figure S5, the optical band gap values of the TNs and Fe-TNs 5% are 3.36 and 3.31 eV, suggesting that Fe doping reduced the optical band gap of TiO_2_. Furthermore, the migration and recombination performance of photoinduced e^−^ and h^+^ was measured via PL measurement. As shown in Figure 6B, the PL intensity of Fe-TNs was reduced obviously compared with that of TNs, demonstrating that the introduction of Fe sites in TNs was favorable for the generation and separation of photogenerated e^−^ and h^+^, thus accelerating the Fe(III)/Fe(II) oxidation–reduction cycle in the Fenton-like reactions. According to the above results, the photo-assisted Fenton-like reaction mechanism for TYL degradation is illustrated in Figure 6C. The photogenerated e^−^ from TiO_2_ migrated along the Ti–O–Fe bonds to the Fe sites. Then, the produced Fe(II) generated from the reduction of Fe(III) could easily react with PMS to generate SO_4_^•−^ and Fe(III). The Fe(III) continued to receive photogenerated e^−^ to complete the Fe(III)/Fe(II) oxidation–reduction cycle. Simultaneously, the photogenerated h^+^, possessing strong oxidation, would also react with TYL directly or oxidize H_2_O to produce •OH to degrade TYL. In conclusion, SO_4_^•−^ •OH, h^+^, and e^−^ are active species involved in TYL degradation. By splitting the C–O, C–N, and C–C bonds in the molecular structure of TYL, CO_2_ and H_2_O were generated to realize the final mineralization and achieve degradation (Figures S6 and S7).

## 3. Materials and Methods

### 3.1. Synthesis of Fe-TNs

The used chemicals of analytical grade were commercially purchased and directly applied without further purification. First, the precursor H_2_Ti_3_O_7_ nanofibers were obtained via hydrothermal treatment of the mixture solution of anatase TiO_2_ powder and NaOH, and subsequently, the H^+^ ion-exchange process. Detailed preparation information for H_2_Ti_3_O_7_ nanofibers is shown in the Supplementary Materials. Subsequently, 1.0 g H_2_Ti_3_O_7_ and a certain amount of FeCl_2_∙4H_2_O (0.2~0.5 g) were put into 200 mL of distilled water. The mixture was exposed to agitation for 2 h, collected via centrifugation, and washed with deionized water three times. After drying, the materials were sealed in a quartz ampoule and heated in a tube furnace at 500 °C for 4 h. The TNs doped with Fe atoms (Fe-TNs) at different ratios were donated as Fe-TNs 2%, Fe-TNs 3%, Fe-TNs 4%, Fe-TNs 5%, and Fe-TNs 6%.

### 3.2. Structural Characterization

XRD (DX2700) was used to survey the composition and the crystalline phase of TNs and Fe-TNs. The surface chemical composition, valence state and chemical bond of Fe-TNs were characterized via X-ray Photoelectron Spectroscopy (XPS, Escalab 250, Thermo Fisher Scientific, Waltham, MA, USA). The morphology and element compositions were surveyed using scanning transmission electron microscopy (STEM, JSM-7001F, Tokyo, Japan). Raman spectra analysis was performed using Thermo Scientific DXR2 equipment at an excitation wavelength of 532 nm. The kinds of radicals were recorded via electron paramagnetic resonance spectroscopy. N_2_ adsorption–desorption experiments were performed using a specific surface area analyzer (BSD-PS2). The optical properties were obtained using a UV-Vis-NIR spectrophotometer (Agilent Cary 5000, Santa Clara, CA, USA). The photoluminescence (PL) spectra were tested using a fluorescence spectrophotometer (FLS9800).

### 3.3. Photocatalytic Fenton-like Degradation for TYL

The catalytic performance of TNs and Fe-TN catalysts for TYL degradation was evaluated in a photocatalytic Fenton-like system. Specifically, 50 mg of materials were weighed and added into 100 mL of TYL solution with an initial concentration of 20 mg/L under dark conditions. Before catalytic reactions took place, the adsorption process proceeded for 1.5 h to reach adsorption equilibrium. Subsequently, 1 mM of PMS was added and the above mixtures were exposed to white LED (300 mW/cm^2^) illumination. The reaction equipment was outfitted with circulating cooling water to keep the solution at 20 °C. In the process of the degradation reaction, 2.0 mL of the samples were taken out at a predetermined time and filtered through a 0.22 μm filter membrane. The remaining TYL concentration in the filtrate was measured via high-performance liquid chromatography (HPLC, EClassical3100). The intermediates of NOR degradation were detected via liquid chromatography (Thermo U3000) and coupled mass spectromery (maXis, Q-TOF, Bruker, Billerica, MA, USA).

## 4. Conclusions

In summary, Fe atom-anchored anatase TiO_2_ nanofibers were fabricated through a hydrothermal process, proton exchange, and an annealing treatment process. The obtained Fe-TN catalyst displayed significantly enhanced TYL degradation performance compared with the pristine TiO_2_ nanofibers in the photocatalytic Fenton-like system. The optimized Fe-TNs-5% catalyst could reach 98.5% TYL degradation efficiency within 4 h. Overall, the introduction of Fe atom sites can extend the light response of TiO_2_ to the visible region and improve the separation of photoexcited electrons and holes. The electrons could transfer to Fe atom sites, realize the Fe(III)/Fe(II) oxidation–reduction cycle, and improve the generation of more SO_4_^•−^ species to degrade TYL. Moreover, the photoinduced holes could be involved in oxidizing TYL, thus significantly enhancing TYL degradation performance efficiency. Therefore, this research proposed a practical strategy to design high-performance and environmentally friendly photocatalytic Fenton-like catalysts for eliminating antibiotic pollution in environmental water.

## Data Availability

All data included in this study are available upon request by contact with the corresponding author.

## Supplementary Materials

The following supporting information can be downloaded at: https://www.mdpi.com/article/10.3390/molecules28196977/s1, Figure S1. (A) N_2_ adsorption and desorption of Fe-TNs; (B) Pore size distribution of Fe-TNs. Figure S2. (A) N_2_ adsorption and desorption of TNs; (B) Pore size distribution of TNs. Figure S3. TEM images of Fe-TNs. Figure S4. TEM images of Fe-TNs 5% after reactions. Figure S5. Tauc plots for TNs and Fe-TNs 5%. Figure S6. LCMS results of TYL in the reactions (0 and 20 min). Figure S7. Degradation pathway of TYL. Table S1. Comparison of several catalysts for degradation of TYL.
