# Supplementary material for: The Use of Iron-Doped Anatase TiO2 Nanofibers for Enhanced Photocatalytic Fenton-like Reaction to Degrade Tylosin
Source: Molecules. 2023 Oct 8;28(19):6977. doi: 10.3390/molecules28196977 (PMC10574164; doi:10.3390/molecules28196977)
Supplement: Supplementary file 1 [file molecules-28-06977-s001.zip › molecules-2625763-supplementary.pdf]

Supplementary Materials

The Use of Iron-Doped Anatase TiO_2 Nanofibers for Enhanced Photocatalytic Fenton-like Reaction to Degrade Tylosin

Xiao Wang [1,2,3,*], Wei Lu [1,2,*], Shangui Zhang [1,2], Changqing Guo [1,2], Kai Yang [1,2], Yan Sun [1,2], Yashi Shao [1,2], Qiyuan Li [1,2], Mingsheng Bu [1,2], Lianfeng Wu [1,2], Bo Wang [1,2] and Dongjiang Yang [3]

[1] State Key Laboratory of Marine Coatings, Qingdao 266071, China; zhangshangui@sinochem.com (S.Z.); wulianfeng@sinochem.com (L.W.)
[2] Marine Chemical Research Institute Co., Ltd., Qingdao 266071, China
[3] School of Environmental Science and Engineering, Qingdao University, Qingdao 266071, China; d.yang@qdu.edu.cn
* Correspondence: wangxiao_qingdao@126.com (X.W.); luwei16@sinochem.com (W.L.)

S1. Experimental section

S1.1 Synthesis and characterization of titanium dioxide nanofibers

In the hydrothermal process, 1 g of anatase TiO_2 powder and 60 mL of 10 M NaOH solution was mixed together. The above mixture was agitated for 12 h and then transferred into a Teflon-lined stainless-steel autoclave. The autoclave was maintained at 180 °C for 48 h. The precipitate (sodium titanate nanofibers, NFs) was recovered, washed with distilled water, exchanged with H^+ (using a 0.1 M HCl solution) to produce $H_2Ti_3O_7$ NFs, and washed again with distilled water. The $H_2Ti_3O_7$ NF product was dried and then calcined in a muffle furnace at 700 °C for 4 h to produce anatase TiO_2 NFs.

S1.2 Measurements

Electron paramagnetic resonance (EPR) tests were performed on a Bruker ELEXSYS E500 X-band spectrometer. Specifically, 10 mg of catalyst was added into 10 mL of water (for •OH and $SO_4^{\bullet -}$ capture), and ultrasonically treated for 5 min. Then, 200 μL of 5,5-dimethyl-1-pyrroline N-oxide (DMPO, 100 mM) was added to the above dispersions (200 μL). Then, the mixture was put into the capillary tube for testing at room temperature. A 300 W Xe lamp with a 420 nm cutoff filter was used as light source. The experiments under dark condition and with light after visible light irradiation for 10 min and 20 min were recorded.

S2. Supplementary figures

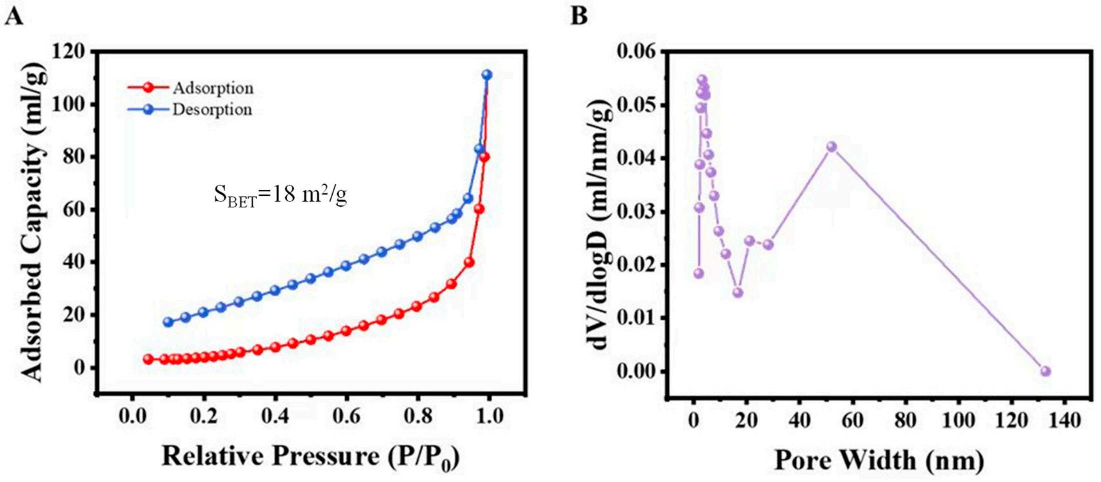

Figure S1. (A) N_2 adsorption and desorption of Fe-TNs; (B) Pore size distribution of Fe-TNs.

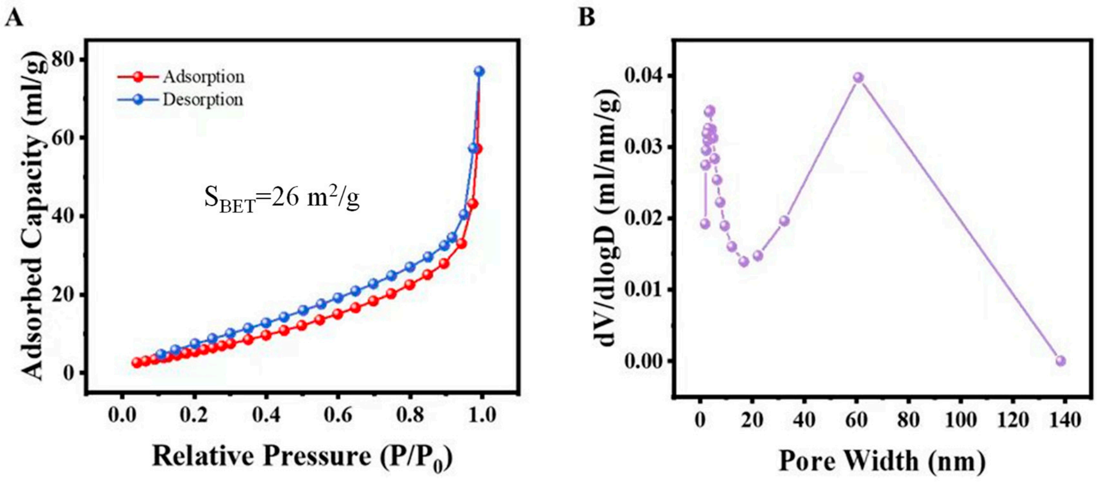

Figure S2. (A) N_2 adsorption and desorption of TNs; (B) Pore size distribution of TNs.

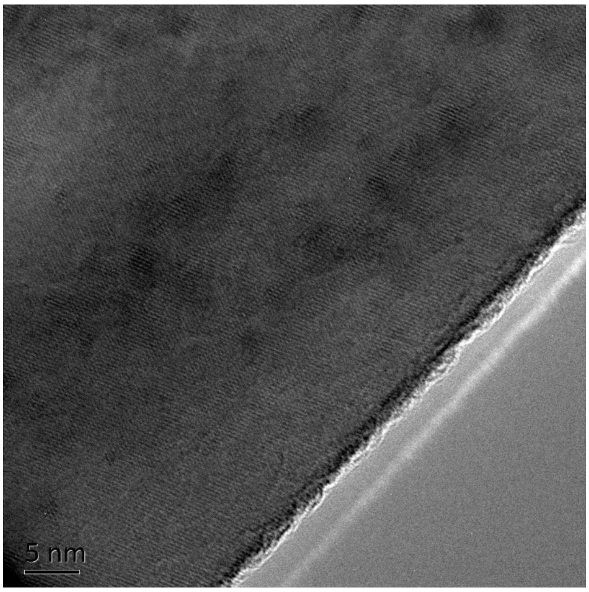

Figure S3. TEM images of Fe-TNs.

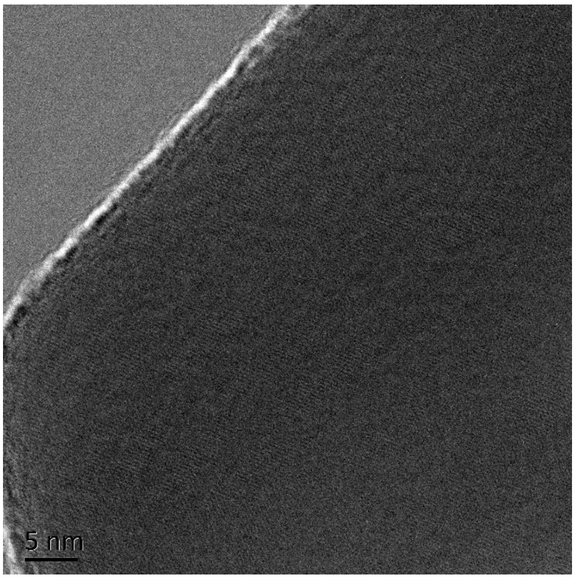

Figure S4. TEM images of Fe-TNs 5% after reactions.

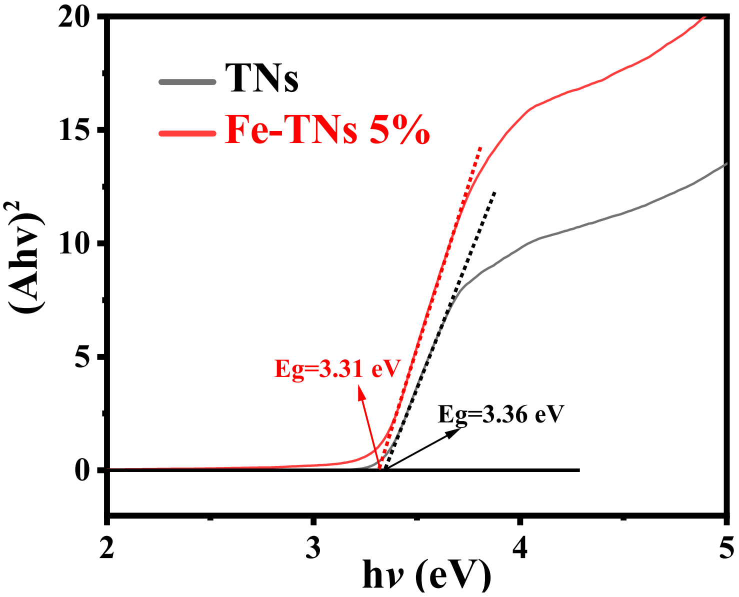

Figure S5. Tauc plots for TNs and Fe-TNs 5%.

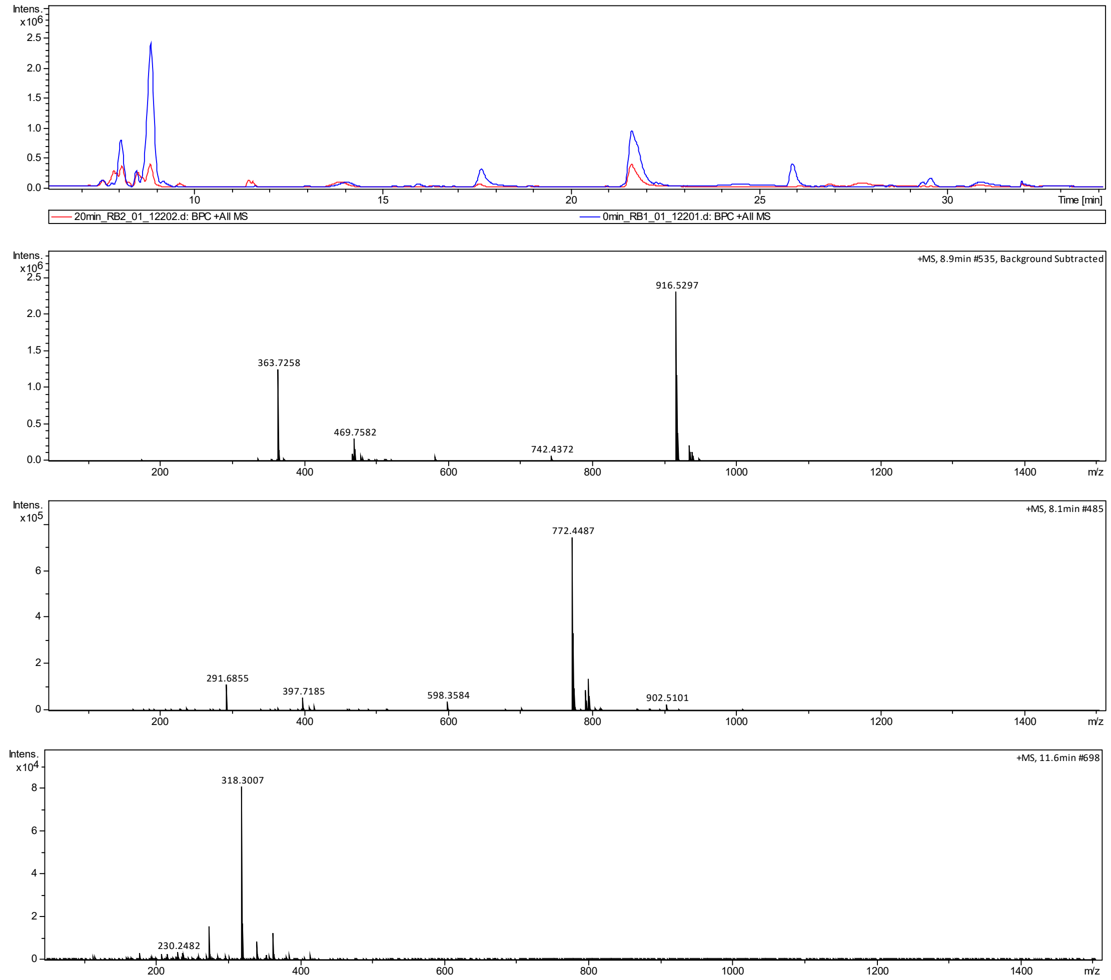

Figure S6. LCMS results of TYL in the reactions (0 and 20 min).

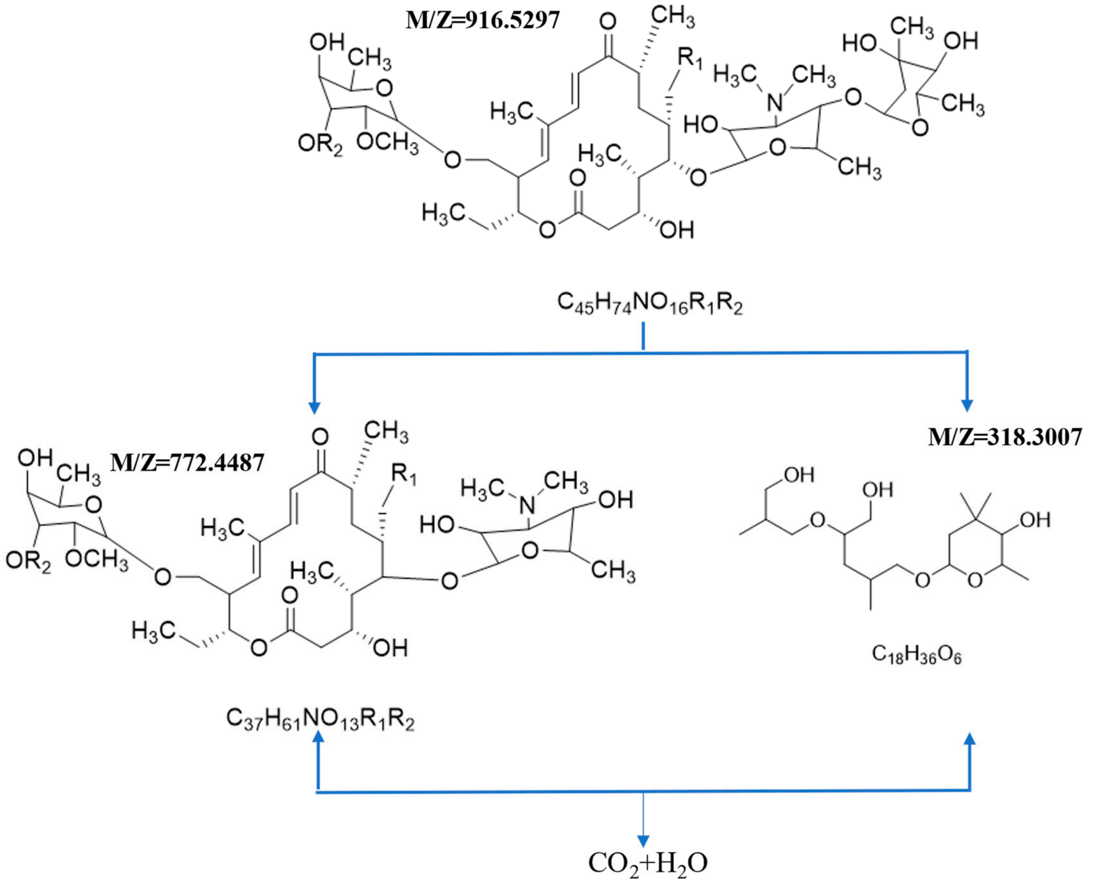

Figure S7. Degradation pathway of TYL.

Table S1. Comparison of several catalysts for degradation of TYL.

Catalysis	Methods	Dosage (g/L)	TYL (mg/L)	Performance (% (min))
ZIF-11(Zn/Co)[1]	Persulfate-activation	0.2	60	80 (30)
Montmorillonite[2]	Photocatalysis	1	30	80.8 (480)
Au/TiO_2-CCBs[3]	Photocatalysis	50	20	92 (180)
TNTAs/P[4]	Photoelectrocatalysis	15*15 mm/(200 mL)	10	79 (250 min)
WS_2@CeO_2 [5]	Sonolysis	1.0	10	75.6 (120 min)
FeS_2/RGO[6]	photocatalysis	1.2	10	90 (120 min)
(This work)	Photocatalysis	0.5	20	98.5 (240 min)

References

[1] Ling, F.; Xiao, X.; Li, Y.; Li, W. A Zn/Co bimetal zeolitic imidazolate framework material as a catalyst to activate persulfates to degrade tylosin in aqueous solutions. New J. Chem., 2022, 46, 18917-18925.

[2] Xu, Y.; Zhang Y.; Wang X.; Wang Z.; Huang, L., Wu, H.; Ren, J., Gu C.; Chen Z. Enhanced photodegradation of tylosin in the presence of natural montmorillonite: Synergistic effects of adsorption and surface hydroxyl radicals. Sci. Total Environ. 2023, 855, 158750.

[3] Zhao, P.; Yang Y.; Pei Y.; Luo X. TEMPO-oxidized cellulose beads embedded with Au-doped TiO_2 nanoparticles for photocatalytic degradation of Tylosin. Cellulose 2023, 30, 1133-1147.

[4] Zhang, G.; Huang, G.; Yang, C.; Chen S., Xu, Y., Zhang, S., Lu, P., Sun, J., Zhu, Y.; Yang, D. Efficient photoelectrocatalytic degradation of tylosin on TiO_2 nanotube arrays with tunable phosphorus dopants. J. Environ. Chem. Eng. 2021, 9, 104742.

[5] Tizhoosh, l. Y.; Khataee, A.; Hassandoost, R.; Soltani, R. D. C.; Doustkhah, E. Ultrasound-engineered synthesis of WS_2@CeO_2 heterostructure for sonocatalytic degradation of tylosin. Ultrason. Sonochem. 2020, 67, 105114.

[6] Zhang, Q.; Li, Y.; Ma, W.; Bai, X.; Ru, X.; Zhang, L.; Zhong, S.; Shu, X. Three-dimensional recyclable FeS_2/reduced graphene oxide aerogel with high porosity reticulated structure for efficient removal of tylosin tartrate. Sep. Purif. Technol. 2023, 324, 124463.
